# Frailty in Internal Medicine Inpatients: Screening and Multidisciplinary Management

**DOI:** 10.7759/cureus.107161

**Published:** 2026-04-16

**Authors:** Sofia Almeida, Pedro M Neves, Vital Da Silva Domingues

**Affiliations:** 1 Internal Medicine, Unidade Local de Saúde de Santo António, Porto, PRT

**Keywords:** clinical screening, frailty, geriatric assessment, hospitalization, internal medicine, multidisciplinary care

## Abstract

Frailty is a clinical syndrome characterized by reduced physiological reserve and increased vulnerability to adverse outcomes. It is highly prevalent among hospitalized patients in Internal Medicine, especially older adults, and is associated with increased risk of complications, functional decline, institutionalization, and mortality. We conducted a narrative literature review across PubMed, Scopus, and Web of Science for studies published until 2025. The review included validated screening tools, inpatient interventions, and their impact on clinical outcomes. Limitations inherent to narrative reviews, including the lack of quantitative synthesis and potential selection bias, are acknowledged. Systematic use of tools such as the Clinical Frailty Scale (CFS) and the FRAIL (Fatigue, Resistance, Ambulation, Illnesses, & Loss of Weight) scale enables rapid and effective identification of frail patients. Targeted inpatient interventions - including medication optimization, nutritional support based on defined criteria, structured early mobilization, and delirium prevention - have shown benefits in reducing length of stay, complications, and functional dependence. Early implementation experiences in Portuguese hospitals suggest feasibility and clinical benefit. Frailty screening and structured management during hospitalization represent a strategic opportunity for Internal Medicine services to improve care efficiency, patient-centered outcomes, and care transitions, with measurable clinical and economic impact.

## Introduction and background

Frailty is a complex clinical condition characterized by reduced physiological reserve and diminished resilience to physiological stressors, leading to increased vulnerability to adverse events such as falls, infections, delirium, functional decline, and mortality [1,2].

To better comprehend frailty, it’s essential to distinguish closely related concepts, such as disability and comorbidity. The World Health Organization (WHO) defines disability as the result of interactions between health conditions and environmental and personal factors, which can occur at three different levels: an impairment in body function or structure, a limitation in activity, or a restriction in participation [3]. Comorbidity refers to any distinct clinical entity that coexists with or occurs during the clinical course of another illness or condition [4].

Although historically associated with geriatric medicine, frailty has become increasingly relevant in Internal Medicine practice due to the progressive ageing of hospitalized populations and the growing burden of multimorbidity and polypharmacy [5].

Epidemiological studies indicate that between 30% and 60% of patients admitted to Internal Medicine wards meet criteria for frailty, depending on the population studied and the screening instrument used [5-7]. Frailty has been consistently associated with prolonged hospital stay, higher rates of early readmission, institutionalization after discharge, and increased healthcare costs [6,7].

Importantly, frailty should not be regarded as an irreversible condition. The current understanding of reverse frailty is a shift from a frail or severely frail state to at least a pre-frail or mildly frail state. Evidence suggests that early identification of vulnerable patients using validated screening tools allows the implementation of targeted multidisciplinary interventions capable of modifying functional outcomes and improving patient prognosis [2,8]. Instruments such as the Clinical Frailty Scale and the FRAIL (Fatigue, Resistance, Ambulation, Illnesses, & Loss of Weight) scale have demonstrated feasibility and prognostic validity in acute care environments [8-11].

This narrative review aims to summarize current evidence regarding frailty screening and multidisciplinary management among Internal Medicine inpatients, with particular emphasis on clinical applicability within hospital settings and the Portuguese healthcare context.

## Review

Methods of the narrative review

A narrative review of the literature was conducted to identify clinically relevant evidence concerning frailty screening and management among Internal Medicine inpatients. The literature search was performed using PubMed, Scopus, and Web of Science databases and included studies published between January 2011 and December 2025. Because several frailty screening instruments were originally described in earlier publications, seminal articles describing these tools were also included, irrespective of publication date.

Search terms included combinations of the descriptors “frailty”, “hospitalization”, “internal medicine”, “screening tools”, “inpatient care”, “geriatric assessment”, and “multidisciplinary care”. Articles were eligible for inclusion if they addressed frailty identification in hospital settings or described interventions targeting frail inpatients and their impact on outcomes such as functional status, length of stay, institutionalization, readmission, or mortality.

Publications focusing exclusively on surgical populations, outpatient settings, or home care were excluded. Recommendations from relevant scientific societies were also reviewed to contextualize the available evidence.

The limitations inherent to narrative reviews are acknowledged, including potential selection bias and the absence of quantitative synthesis. However, this approach allows the integration of heterogeneous evidence into a clinically meaningful framework applicable to Internal Medicine practice.

Frailty screening tools in the hospital setting

Frailty has a high prevalence among Internal Medicine inpatients and represents an important predictor of adverse hospital outcomes, including mortality, functional decline, and institutionalization [5-7]. Early recognition of frailty is therefore essential to guide targeted clinical interventions.

Among the screening instruments evaluated in the literature, three have demonstrated particular applicability in hospital settings: the Clinical Frailty Scale, the FRAIL scale, and PRISMA (Program of Research to Integrate the Services for the Maintenance of Autonomy)-7 [8-10].

The Clinical Frailty Scale is a nine-point clinical assessment tool based primarily on the patient’s baseline functional status and comorbidity burden. It allows rapid classification of patients according to frailty severity and can usually be applied in less than one minute. Several studies have demonstrated that the Clinical Frailty Scale correlates strongly with mortality, length of hospital stay, institutionalization, and functional decline among hospitalized older adults [8,11]. Because of its simplicity and prognostic value, it has become one of the most widely used frailty screening tools in acute care environments.

The FRAIL scale consists of a brief five-item questionnaire evaluating fatigue, resistance, ambulation, illness burden, and weight loss. Its simplicity allows rapid screening during hospital admission and facilitates early identification of patients at risk of frailty. Studies evaluating its predictive performance have demonstrated associations with disability, hospitalization, and mortality [9,12].

PRISMA-7 is a seven-item questionnaire originally developed for community-based case finding of disability and frailty among older adults. Although the tool demonstrates high sensitivity, its specificity is lower in hospital settings, and it remains more commonly used in primary care screening programs rather than acute inpatient evaluation [10,13].

Together, these instruments provide complementary approaches for identifying frailty in hospitalized patients (see Appendices). Despite their usefulness, there is some controversy regarding the applicability of these tools, namely the absence of a universally optimal screening tool, the debate between universal and selective screening, the limited evidence in patients under 65 years, and ethical considerations regarding the use of frailty in clinical decision-making.

In Portugal, experiences with the systematic use of the Clinical Frailty Scale have demonstrated its clinical applicability in hospitalized older patients [14]. Pereira Pinto et al. described the translation and validation of the CFS into European Portuguese and demonstrated its integration with reference hospital services in northern Portugal, including the Centro Hospitalar Universitário São João, Porto [14]. These findings support the feasibility of implementing structured frailty screening and highlight its potential to inform individualized care plans in Portuguese hospital settings [14].

Although still limited, these national data suggest that structured frailty management is feasible, effective, and potentially sustainable in the hospital setting and should be expanded systematically [14].

 The literature supports that frailty is a prevalent, clinically relevant condition with a direct impact on the recovery trajectory of internal medicine inpatients [1,2,6]. Its association with adverse outcomes justifies the systematic adoption of screening and intervention strategies during hospitalization [5,7]. The high prevalence, combined with the often-reversible nature of frailty, makes it a priority clinical target [2,11].

The Clinical Frailty Scale emerges as the most suitable tool for the Portuguese hospital context, combining simplicity, prognostic validity, and good acceptance by clinical teams [11,14]. However, its integration into clinical practice remains limited due to the absence of standardized institutional protocols and a lack of specific training [15]. Additionally, hospital workload and the perception of frailty as a “geriatric problem” contribute to its under-recognition by internal medicine clinicians [2].

International experiences, such as the UK’s Comprehensive Geriatric Assessment (CGA) model, provide consolidated examples [16]. In NHS hospitals, CGA is implemented transversally with dedicated multidisciplinary teams, clear functional targets, and quality indicators related to frailty [16]. While resource-intensive, the underlying philosophy is replicable. In Portugal, adaptation should consider local availability, progressive integration in pilot services, and targeted training for generalist internists.

Inpatient interventions

Regarding interventions, the most robust studies demonstrated consistent benefits from multidimensional measures (see Appendices) applied during hospitalization [2,6,7]. Pharmacological optimization, based on medication review using STOPP/START (Screening Tool of Older Persons' Potentially Inappropriate Prescriptions/Screening Tool to Alert to Right Treatment) criteria and deprescribing high-risk drugs (e.g., benzodiazepines, anticholinergics), was associated with reduced adverse events and delirium [7].

Nutritional status is another key determinant of frailty progression. Undernutrition and sarcopenia are common among hospitalized older adults and contribute to functional decline and delayed recovery. Early nutritional risk screening using validated instruments such as the Mini Nutritional Assessment (MNA-SF) and Nutritional Risk Screening 2002 (NRS-2002) enables timely identification of patients requiring nutritional intervention and supplementation [17,18].

Early mobilization, implemented with physiotherapy support within the first 48 hours, reduced immobilization syndromes and improved autonomy at discharge [2].

Delirium prevention had a significant impact on frail patients, especially when simple measures were applied, such as orientation to time and place, appropriate lighting, correction of visual and auditory deficits, and minimization of night-time stimuli [7]. Coordination with continuing care, with discharge planning initiated in the first days of admission, facilitated transition to convalescence or rehabilitation units with measurable clinical and social benefits [19,20].

Implementation barriers and health-system context

Interventions such as medication review, individualized nutritional support, early physiotherapy, and delirium prevention have shown consistent benefits [2,6,7]. However, implementation faces several barriers: limited human resources, poor service coordination, insufficient time for structured functional assessment, lack of specific funding, and resistance to organizational change [15]. Overcoming these challenges requires institutional leadership, dedicated clinical teams, and recognition of functionality as both a clinical and administrative outcome in hospitals [14,15].

In this context, the internist plays a strategic role: as the clinician most consistently involved in complex patient care, best positioned to coordinate individualized plans, and the main link to continuity of care [2]. Frailty should no longer be regarded as a passive risk factor but as a functional clinical warning sign with direct implications for medical management, service organization, and health outcomes [2,11].

Implications for clinical practice

Integrating frailty screening into internal medicine departments should be a clinical and organizational priority [11,14]. Systematic application of the Clinical Frailty Scale at admission, complemented by targeted interventions and well-defined protocols - including medication review, structured nutritional support, early mobilization, and delirium prevention - can improve functional trajectory and prognosis [2,6,7].

Operationalising this approach requires team-specific training, coordination with physiotherapy, nutrition, pharmacy, and social services, and institutional commitment to functionality as a clinical outcome [14]. Including frailty indicators in discharge reports and monitoring them as performance metrics can enhance the sustainability and visibility of these interventions.

In this model, the internist serves as the central coordinator of care for the frail patient, integrating services, prioritising functionality, and promoting therapeutic decisions tailored to individual recovery potential [2].

## Conclusions

Frailty represents a frequent and clinically significant condition among patients admitted to Internal Medicine wards and is strongly associated with adverse outcomes, including hospital readmission, functional decline, institutionalization, and mortality. Early identification through validated screening tools helps clinicians to recognize vulnerable patients and initiate targeted multidisciplinary interventions with demonstrated clinical benefits.

Recognizing frailty as a central clinical concept in the care of hospitalized patients, therefore, represents an important step toward improving patient-centered care, optimizing healthcare resources, and strengthening continuity of care across healthcare systems.

## Appendices

**Table 1:** Frailty screening tools and interventions during hospitalization MNA: Mini-Nutritional Assessment, NRS: Nutritional Risk Screening

Tool/intervention	Key characteristics	Clinical applicability	Key references
Clinical Frailty Scale (CFS)	9-point scale based on baseline functional status	High – recommended in guidelines and hospital practice	[6,9,16]
FRAIL scale	Five-item questionnaire assessing fatigue, resistance, ambulation, illness burden, and weight loss	Moderate – useful at admission and for initial triage	[7,10]
PRISMA-7	Seven-item frailty screening questionnaire	Low – more suitable for outpatient/community settings	[8,11]
Medication optimization	Structured medication review and deprescribing	High – priority intervention in frail patients	[2]
Nutritional assessment (MNA / NRS-2002)	Early identification of malnutrition risk and supplementation	High – essential for patients at nutritional risk	[12,13]
Early mobilization	Physiotherapy and early functional recovery strategies	High – early intervention associated with better outcomes	[2,18]
Delirium prevention	Orientation support, sleep hygiene, sensory aids	Moderate – especially useful in older patients with cognitive impairment	[6]
